# Knowledge, attitude, and practice regarding dengue virus infection among inhabitants of Aceh, Indonesia: a cross-sectional study

**DOI:** 10.1186/s12879-018-3006-z

**Published:** 2018-02-27

**Authors:** Harapan Harapan, Yogambigai Rajamoorthy, Samsul Anwar, Aslam Bustamam, Arsil Radiansyah, Pradiba Angraini, Riny Fasli, Salwiyadi Salwiyadi, Reza Akbar Bastian, Ade Oktiviyari, Imaduddin Akmal, Muhammad Iqbalamin, Jamalul Adil, Fenni Henrizal, Darmayanti Darmayanti, Rovy Pratama, Abdul Malik Setiawan, Mudatsir Mudatsir, Panji Fortuna Hadisoemarto, Mandira Lamichhane Dhimal, Ulrich Kuch, David Alexander Groneberg, Allison Imrie, Meghnath Dhimal, Ruth Müller

**Affiliations:** 1 Medical Research Unit, School of Medicine, Syiah Kuala University, Banda Aceh, Indonesia; 2 Tropical Disease Centre, School of Medicine, Syiah Kuala University, Banda Aceh, Indonesia; 3 Department of Microbiology, School of Medicine, Syiah Kuala University, Banda Aceh, Indonesia; 4 School of Biomedical Sciences, The University of Western Australia, Nedlands, Australia; 5 Universiti Tunku Abdul Rahman, Selangor, Malaysia; 6 Department of Statistics, Faculty of Mathematics and Natural Sciences, Syiah Kuala University, Banda Aceh, Indonesia; 7 Department of Biology, Faculty of Teacher Training and Education, Syiah Kuala University, Banda Aceh, Indonesia; 8 Department of Microbiology, Medical Faculty, Maulana Malik Ibrahim State Islamic University, Malang, Indonesia; 9 Department of Public Health, Faculty of Medicine, Padjadjaran University, Bandung, Indonesia; 10 Institute of Occupational Medicine, Social Medicine and Environmental Medicine, Goethe University, Frankfurt am Main, Germany; 11 Nepal Health Research Council (NHRC), Ministry of Health Complex, Kathmandu, Nepal

**Keywords:** Dengue, Dengue fever, Knowledge, Attitude, Practice, KAP

## Abstract

**Background:**

The Indonesian region of Aceh was the area most severely affected by the earthquake and tsunami of 26 December 2004. Department of Health data reveal an upward trend of dengue cases in Aceh since the events of the tsunami. Despite the increasing incidence of dengue in the region, there is limited understanding of dengue among the general population of Aceh. The aim of this study was to assess the knowledge, attitude, and practice (KAP) regarding dengue among the people of Aceh, Indonesia in order to design intervention strategies for an effective dengue prevention program.

**Methods:**

A community-based cross-sectional study was conducted in Aceh between November 2014 and March 2015 with a total of 609 participants living in seven regencies and two municipalities. Information on the socio-demographic characteristics of participants and their KAP regarding dengue was collected using a pre-tested structured questionnaire. The KAP status (good vs. poor) of participants with different socio-demographic characteristics was compared using Chi Square-test, ANOVA or Fisher’s exact test as appropriate. Logistic regression analysis was used to determine the predictors of each KAP domain.

**Results:**

We found that 45% of participants had good knowledge regarding dengue and only 32% had good attitudes and good dengue preventive practices. There was a significant positive correlation between knowledge and attitudes, knowledge and practice, and attitudes and practice. In addition, people who had good knowledge were 2.7 times more likely to have good attitudes, and people who had good attitudes were 2.2 times more likely to have good practices regarding dengue. The level of education, occupation, marital status, monthly income, socioeconomic status (SES) and living in the city were associated with the knowledge level. Occupation, SES, and having experienced dengue fever were associated with attitudes. Education, occupation, SES and type of residence were associated with preventive practices.

**Conclusion:**

Our study suggests that dengue prevention programs are required to increase KAP levels regarding dengue in the communities of Aceh.

**Electronic supplementary material:**

The online version of this article (10.1186/s12879-018-3006-z) contains supplementary material, which is available to authorized users.

## Background

Dengue fever (DF), caused by infection with any of the four dengue virus (DENV) serotypes, has become the most important mosquito-borne viral disease in humans [1]. Dengue fever is associated with significant morbidity, mortality, and economic cost, particularly in developing countries [2]. Since DF was first documented in Indonesia’s capital Jakarta in 1968, it has become prevalent in all provinces of the country and is now a major public health problem [3]. Nearly 60% of the Indonesian population (240 million) live in areas where DENV is known to be circulating. In 2016 there were 201,885 notified cases of DENV infections (77.96 per 100,000 population) and 1585 deaths due to DF [4].

Aceh, located at the northern end of Indonesia’s Sumatra Island, was the most severely affected area by the earthquake and tsunami disaster of 26 December 2004. In 2005, the WHO warned of an increased DF risk in tsunami-affected areas [5]. Reports issued between 2003 and 2011 showed an upward trend of reported DF cases in Aceh [6]. In 2003, before the earthquake and tsunami, the incidence of DF was 2.76 per 100,000. It increased significantly to 35.36 per 100,000 in 2009, and again to 56.40 per 100.000 in 2011 [6]. Recently, in 2016, a total of 2651 DF cases were reported in Aceh (52.02 per 100,000 population) [4].

Dengue prevention and control programme has been placed in national scale by Ministry of Health of Indonesia through Directorate General for Communicable Diseases Control since 1968 with the main objective is to prevent and reduce dengue morbidity and mortality at family and community levels [7]. In 1970s, Indonesia started to implement the peri-focal spraying strategy and health education in a limited area and in 1980, in addition to peri-focal spraying, mass larviciding was adopted [7]. In 1992, organized community efforts were conducted at the village level through the Dengue Hemorrhagic Fever Working Group. This group included one member from Women Empowerment Welfare Group. In the same year, a series of law and legislations of Dengue Prevention and Control Programme were issued. Since 2000, the strategy of dengue control programme has been focused on community participation in source reduction of breeding places [7].

Despite the increasing incidence of DF in Aceh there has been no study to assess the knowledge, attitude and practice (KAP) of Aceh communities regarding DENV transmission and its prevention. Therefore, the aim of the present study was to assess and compare the KAP among community groups in Aceh, in order to design intervention strategies for an effective dengue prevention program.

## Methods

### Study design and setting

**Fig. 1:**
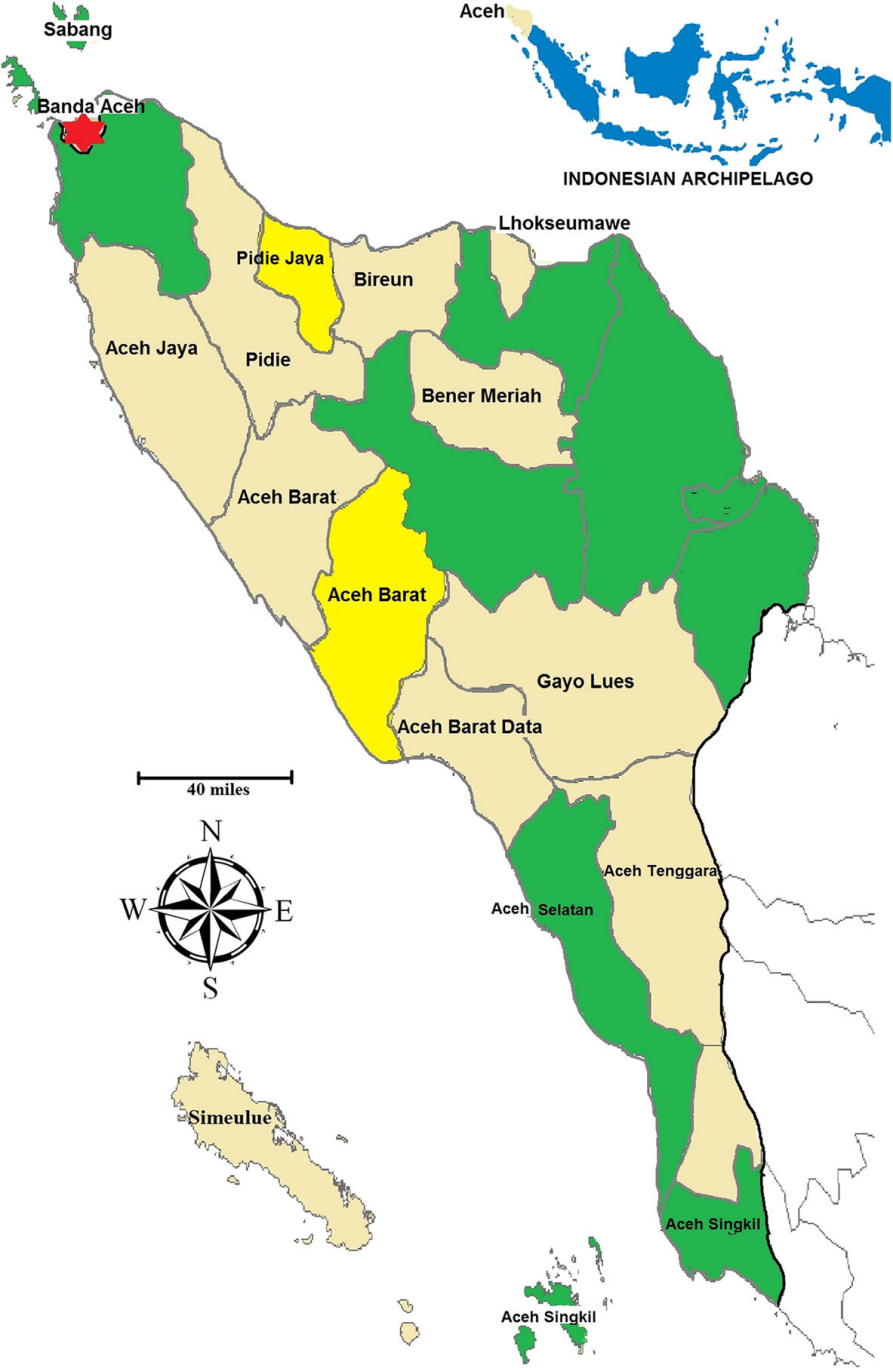
Study areas (green) and areas of questionnaire pre-testing (yellow) in Aceh province, Indonesia

A cross-sectional study was conducted in the province of Aceh, which is located in the westernmost region of the Indonesian archipelago and has a surface area of 57,956 km^2^. In 2014, Aceh had a total population of 4,791,924 in 18 regencies and 5 municipalities [8]. This study included localities in the southwestern (from 0 to 25 m above sea level), central (~ 1200 m above sea level) and northern (25 to 100 m above sea level) regions of Aceh (Fig. 1). The study was conducted in seven regencies (Aceh Tengah, Aceh Besar, Aceh Utara, Aceh Singkil, Aceh Timur, Aceh Selatan and Aceh Tamiang) and two municipalities (Langsa and Sabang) of Aceh. A reliability test of questionnaires was separately conducted among populations in two additional regencies (Aceh Barat Daya and Aceh Pidie Jaya) (Fig. 1). Data were collected from November 2014 to March 2015. The design, setting, analyses and reporting of this study adhered to the STROBE guidelines for cross-sectional studies in epidemiology (see Additional file 1 for the detailed checklist of STROBE criteria [9]).

### Sampling and sample size

So far no data related to the KAP towards dengue in Aceh have been available. Therefore, to calculate a representative sample size for the Aceh population (4,791,924), we assumed that 50% of participants would have good KAP regarding dengue. With a 5% margin of error and 95% confidence level, 385 participants were required to achieve the minimum recommended sample size. To recruit the samples, seven out of 18 regencies and two out of five municipalities in Aceh were selected randomly. The minimum number of participants from each study site was 45. To minimize study design effect and to obtain more robust statistical power, a minimum of 60 participants were required from each study site. However, for regencies with a high population density, a higher number of participants was allocated (i.e. additional 10% to 50% number of participants was allocated compared to the regency with the low population density). In addition, two regencies were selected randomly for the questionnaire reliability test. All inhabitants who were aged over 16 years, had resided in the specified regency or municipality for more than 3 months, and were able to communicate were considered to be eligible for inclusion as participants of the study.

### Study instrument

To facilitate the interviews, a set of validated and pre-tested questionnaires, consisting questions related to asset index [10] and KAP regarding DF [11–13] was used. Before the questionnaire was used in the study, it was tested for internal consistency among 52 participants in two regencies. The data from these participants were not included in the final analysis. A minimum of Cronbach’s Alpha of 0.7 was considered to reflect acceptable internal reliability [14].

### Study variables

#### Explanatory variables

We collected data on the age, education, occupation, religion, marital status, income, and type of residence of participants, and whether or not they or family members had already suffered from DF. The participants were also asked regarding their source of information on DF. The asset index from D Filmer and L Pritchett [10] was adapted to measure and categorize the socioeconomic status (SES) of participants. This asset index has been modified for the Indonesian and current contexts [13]. It measured the ownership of 15 household asset and the full list of household assets has been published elsewhere [15]. The ownership of one asset item was given a score of one, and its absence a score of zero (i.e. the minimum and maximum asset score was 0 and 15, respectively). For each household, the asset index was constructed as the sum of standardized asset scores multiplied by their respective factor loadings [13]. Finally, quintiles of the asset index were calculated and households classified into 1st to 5th quintile wherein the 1st quintile is the poorest and the 5th the least poor.

#### Response variables

Knowledge regarding DF consisted of two parts, namely knowledge about symptoms and signs of DF, and knowledge about DENV transmission. A total of 28 questions adapted from previous studies were used to measure this domain [11, 12]. Possible responses to all of the questions were “yes” or “no”; there was no “do not know” option. A correct response was given a score of one, whereas an incorrect response was given a score of zero. Participant knowledge was calculated as the total sum of correct responses so that higher scores indicated better knowledge about DF. Attitude towards DF was measured using a set of 15 questions adapted from previous studies [11–13]. Participants were asked to respond to the questions on a five-point Likert-like scale as follows: 1 = Strongly disagree; 2 = Somewhat disagree; 3 = Neither agree nor disagree; 4 = Somewhat agree; and 5 = Strongly agree. A high score was given when the statement and the alternative answer defined positive attitudes. The attitude score was computed as the sum of participant responses. Preventive measures against DENV infection was measured using 16 questions adopted from previous studies [11, 12]. This domain included measures such as preventing mosquito-man contact and eliminating mosquito breeding place. Each correct response was given a score of one, whereas an incorrect response was given a score of zero. Hence, higher scores indicate better preventive practice.

### Interview and data collection

To collect the interested information from inhabitants, face-to-face interviews were conducted in Bahasa Indonesia by data collection team. All member of data collection team were medical doctors and a brief training was provided prior to actual study. To avoid the bias, the correct answers to the survey questions were not provided. Prior to interview, an overview of the study aims, risks and benefits was given and explained to potential participants. Those who agreed to participate were asked to sign an informed consent form. Each informed consent form and its matching questionnaire was assigned a three-digit identifier. The latter was used in all analyses. Once informed consent was obtained, the interviewers conducted a structured interview assisted by a validated questionnaire. Participation in this study was voluntary and no incentive was given to the participants. Participants could stop and leave the study at any time during the interview.

### Statistical analysis

The KAP assessment was executed using a scoring system. Scores for each question within a domain were summed up to arrive at a single value out of a total score of 28, 75 and 16 for the respective KAP domains. Participants’ KAP levels were defined as “good” or “poor” based on an 80% cut-off point [11]. To determine the role of socio-demographic characteristics on KAP, differences in socio-demographic status were compared with the KAP levels (good and poor) using the Chi Square-Test, ANOVA or Fisher’s exact test as appropriate. Logistic regression analysis was used to determine the predictors of each KAP domain. In the univariate logistic regression, all explanatory variables were included. Levels of KAP, “good” vs. “poor”, were used as the outcome variables in the logistic regressions. In the next step, significant explanatory factors from univariate analysis (*p* ≤ 0.25) were entered into the multivariate analysis. Confounding factors were explored by comparing the difference between the adjusted odds ratio (aOR) in multivariate analyses and the crude odds ratio (OR) in univariate analyses, of a particular predictor variable on the KAP domain.

The correlation values among KAP scores and between KAP score and asset index were calculated using Spearman’s rank correlation (r_s_). This correlation was chosen because the KAP scores were not normally distributed as revealed by the Kolmogorov–Smirnov test. In order to calculate confidence intervals for Spearman’s rank correlation, the procedure by DG Bonett and TA Wright [16] was followed. All analyses were performed using Statistical Package for the Social Sciences software (SPSS for Windows, Version 15, Chicago, IL).

## Results

### Questionnaire validity test

The internal consistency of the questionnaire was confirmed using 52 interviews with participants from two different study sites (Aceh Barat Daya and Aceh Pidie Jaya) that have socio-demographic backgrounds similar to the main study participants. The Cronbach’s Alpha coefficient of KAP domain was 0.704, 0.962 and 0.720, respectively. Details of questions used to assess the KAP domain and the distribution of correct responses among participants are presented in Additional files 2, 3 and 4, respectively.

### Study population characteristics

The data presented in this study was a part of Aceh Dengue Study and the characteristics of the research participants, in part, have been described elsewhere [15, 17–20]. Briefly, for this specific study, 677 healthy community members were surveyed and 68 (10.0%) participants were excluded from the analysis due to missing information. A total of 609 inhabitants, who provided data for all sections of questionnaire, were included in the final analysis (Table 1). Of the total participants, 70.3% were female and more than half (54.0%) were 17–29 years old. More than half of participants (51.4%) had diploma a certificate or a university degree. The majority (68.1%) were living in suburban areas with more than half (51.4%) of them earned less than 1 million Indonesian Rupiah (approximately US$ 81) per month. Although less than one-tenth of the participants included in this study reported having had an episode of DF, 22.2% of the participants declared had family member(s) who had suffered from DF. This indicates a high prevalence of DF in Aceh.

**Table 1:** Characteristics of study participants (*n* = 609)

Variable	*n* (%)	Mean score		
		Knowledge (SD)	Attitude (SD)	Practice (SD)
Regency				
Aceh Tengah	66 (10.8)	17.5 (4.89)	56.6 (6.41)	10.6 (3.61)
Langsa	74 (12.2)	19.2 (3.57)	28.1 (4.90)	10.9 (2.79)
Aceh Besar	90 (14.8)	21.3 (2.61)	61.8 (5.67)	12.1 (2.64)
Aceh Utara	60 (9.9)	21.5 (2.90)	61.3 (8.49)	12.0 (3.22)
Aceh Singkil	57 (9.4)	21.2 (3.16)	61.8 (5.89)	12.4 (2.48)
Sabang	60 (9.9)	23.0 (2.46)	62.2 (7.55)	12.7 (2.33)
Aceh Timur	60 (9.9)	22.7 (3.83)	63.6 (6.87)	12.5 (2.74)
Aceh Selatan	82 (13.5)	20.1 (3.47)	54.3 (10.53)	11.9 (2.84)
Aceh Tamiang	60 (9.9)	21.5 (3.94)	57.5 (11.66)	12.0 (2.60)
Age group (years)				
17–29	329 (54.0)	21.2 (3.5)	55.2 (14.14)	11.9 (2.89)
30–44	206 (33.8)	20.6 (4.3)	57.3 (12.00)	12.1 (2.94)
45–59	65 (10.7)	19.9 (3.7)	55.7 (12.59)	11.7 (2.67)
60–84	9 (1.5)	19.8 (2.6)	55.0 (13.37)	12.1 (3.62)
Sex				
Male	181 (29.7)	20.7 (4.01)	54.6 (13.89)	11.7 (2.90)
Female	428 (70.3)	20.9 (3.72)	56.5 (12.99)	12.0 (2.89)
Education				
Illiterate	15 (2.5)	18.5 (4.24)	51.9 (14.60)	9.4 (3.07)
Primary school	27 (4.4)	18.1 (3.92)	52.4 (9.89)	9.9 (3.27)
Junior high school	35 (5.7)	17.1 (4.48)	48.5 (13.23)	10.1 (3.37)
Senior high school	219 (36.0)	20.3 (3.57)	54.8 (13.60)	11.7 (2.82)
Diploma	138 (22.7)	21.6 (3.25)	56.8 (13.42)	12.6 (2.82)
Graduated	175 (28.7)	22.4 (3.34)	58.9 (12.30)	12.6 (2.39)
Occupation				
Farmer	157 (25.8)	21.9 (3.44)	57.7 (13.44)	12.6 (2.18)
Civil servant	94 (15.4)	21.9 (3.40)	56.5 (14.02)	13.1 (2.46)
Private employee	121 (19.9)	20.3 (3.76)	55.6 (12.96)	12.0 (2.94)
Entrepreneur	87 (14.3)	17.7 (4.06)	55.2 (9.33)	10.1 (3.34)
Student/University student	150 (24.6)	21.4 (3.24)	54.4 (14.69)	11.4 (2.92)
Religion				
Muslim	601 (98.7)	20.9 (3.81)	56.1 (13.16)	11.9 (2.90)
Other	8 (1.3)	19.4 (4.10)	45.0 (18.22)	11.6 (1.92)
Marital Status				
Unmarried	258 (42.4)	21.5 (3.21)	55.2 (14.33)	11.9 (2.93)
Married	333 (54.7)	20.4 (4.20)	56.3 (12.50)	12.0 (2.88)
Widowed	18 (3.0)	21.4 (3.03)	58.2 (11.73)	11.4 (2.70)
Monthly income (IDR)				
< 1 million	313 (51.4)	20.3 (3.94)	55.0 (13.76)	11.6 (3.09)
1 – ≤ 2 million	124 (20.4)	20.5 (3.55)	54.6 (13.63)	11.9 (2.85)
2 – ≤ 3 million	96 (15.8)	21.9 (3.38)	57.7 (12.64)	12.1 (2.71)
> 3 million	76 (12.5)	22.4 (3.53)	59.5 (10.56)	13.1 (1.89)
Type of residence				
City	194 (31.9)	21.8 (3.24)	55.9 (14.75)	12.3 (2.64)
Suburb	415 (68.1)	20.4 (4.99)	55.9 (12.55)	11.7 (2.99)
Family member(s) suffered from dengue fever				
Yes	135 (22.2)	21.0 (3.34)	54.3 (15.43)	12.1 (2.71)
No	474 (77.8)	20.8 (3.94)	56.4 (12.58)	11.9 (2.94)
Personally experienced dengue fever				
Yes	56 (9.2)	21.6 (3.52)	54.9 (16.18)	11.8 (3.11)
No	553 (90.8)	20.8 (3.83)	56.0 (12.96)	11.9 (2.87)
Socioeconomic status				
Poorest quintile	122 (20.0)	18.9 (4.22)	56.8 (11.95)	10.8 (3.37)
2nd	123 (20.2)	20.8 (4.03)	55.5 (13.70)	11.6 (2.85)
3rd	122 (20.0)	20.5 (3.29)	55.5 (14.15)	11.7 (2.80)
4th	121 (19.9)	21.2 (3.48)	55.8 (14.38)	12.2 (2.54)
Richest quintile	121 (19.9)	22.7 (3.26)	60.0 (11.17)	13.0 (2.31)

*IDR* Indonesian rupiah, *SD* standard deviation

### Knowledge about signs and symptoms of dengue fever and transmission of dengue virus

A statistically significant difference in the mean knowledge score was identified between regencies (*P* < 0.001). The highest mean knowledge score was achieved in Sabang and the lowest in Aceh Tengah (Table 1). Out of total participants, 280 of them (45.9%) had a good knowledge level. Factors associated with good knowledge were high education level, working as a civil servant, unmarried status, high monthly income, high SES and living in the city (*P* < 0.05). Age group, sex and religion had no association with participants’ knowledge (Table 2).

**Table 2:** Univariate and multiple logistic regression analysis showing predictors of knowledge levels (good vs. poor) (*n* = 609)

Independent variable	Univariate		Multivariate	
	OR (95% CI)	*P-*value	aOR (95% CI)	*P*–value
Regency		< 0.001	–	*–*
Aceh Besar	1			
Aceh Tengah	0.26 (0.12–0.55)			
Langsa	0.35 (0.17–0.68)			
Aceh Utara	1.16 (0.60–2.24)			
Aceh Singkil	1.13 (0.58–2.19)			
Sabang	4.86 (2.24–10.55)			
Aceh Timur	2.18 (1.11–4.30)			
Aceh Selatan	0.45 (0.24–0.85)			
Aceh Tamiang	1.33 (0.69–2.57)			
Age group (years)		0.159		0.639
17–29	1		1	
30–44	0.85 (0.60–1.21)		0.97 (0.55–1.70)	
45–59	0.61 (0.35–1.05)		0.65 (0.28–1.51)	
60–84	0.29 (0.06–1.45)		0.48 (0.07–3.09)	
Sex		0.144		0.215
Male	1		1	
Female	1.29 (0.91–1.84)		1.29 (0.86–1.94)	
Education		< 0.001		0.002
Illiterate	1		1	
Primary school	1.85 (0.32–10.61)		1.80 (0.28–11.57)	
Junior high school	1.08 (0.18–6.32)		0.66 (0.10–4.41)	
Senior high school	3.52 (0.77–16.02)		1.44 (0.27–7.67)	
Diploma	6.88 (1.49–31.67)		2.34 (0.42–12.78)	
Graduated	13.81 (3.01–63.29)		3.95 (0.72–21.63)	
Occupation		< 0.001		0.155
Farmer	1		1	
Civil servant	8.84 (4.45–17.58)		1.40 (0.75–2.9	
Private employee	8.81 (4.22–18.37)		1.06 (0.57–1.99)	
Entrepreneur	4.25 (2.09–8.64)		0.47 (0.63–1.19)	
Student/University student	5.76 (2.89–11.48)		1.39 (0.10–3.04)	
Religion		0.248		0.585
Muslim	1		1	
Other	0.38 (0.07–1.93)		0.612 (0.10–3.57)	
Marital status		0.045		0.856
Unmarried	1		1	
Married	0.67 (0.48–0.93)		0.96 (0.55–1.66)	
Widowed	1.19 (0.45–3.12)		1.75 (0.50–6.14)	
Monthly income (IDR)		< 0.001		0.073
< 1 million	1		1	
1 – ≤ 2 million	0.85 (0.55–1.31)		0.89 (0.53–1.50)	
2 – ≤ 3 million	2.39 (1.50–3.83)		1.74 (0.92–3.28)	
> 3 million	3.06 (1.80–5.20)		2.14 (1.03–4.41)	
Socioeconomic status		< 0.001		0.025
Poorest quintile	1		1	
2nd	1.62 (0.94–2.77)		0.93 (0.50–1.71)	
3rd	2.23 (1.31–3.80)		1.72 (0.95–3.12)	
4th	2.63 (1.54–4.48)		1.39 (0.75–2.58)	
Richest quintile	5.24 (3.03–9.06)		2.13 (1.10–4.11)	
Type of residence		< 0.001		0.610
City	1		1	
Suburb	0.51 (0.36–0.72)		0.76 (0.40–1.43)	
Family member(s) suffered from dengue fever		0.989		
Yes	1		–	–
No	1.00 (0.68–1.47)			
Personally suffered from dengue fever				
Yes	1	0.081	1	0.402
No	0.61 (0.35–1.06)		0.75 (0.60–1.42)	

*aOR* Adjusted odds ratio, *IDR* Indonesian rupiah

Our study identified increased odds of having good knowledge if the participants had a diploma certificate or graduated from university compared to participants who were illiterate (Table 2). Higher monthly income and higher SES were also significantly associated with good knowledge (*P* ≤ 0.001). In addition, participants living in the cities were approximately twice as likely to have good knowledge compared to participants living in the suburbs. Interestingly, having personally experienced DF, or having a family member with a history of DF, was not associated with an increase in the participants’ knowledge.

After excluding insignificant predictor factors (*P* > 0.25) from the analysis, the multivariate model revealed that SES was the only independent predictor factor of knowledge regarding DF (Table 2). In the final model, there was increased odds of having good knowledge among participants who were classified in the richest quintile, compared to the poorest (1st quintile) with OR: 2.13 with 95% confidence interval (95% CI): 1.10–4.11 (Table 2).

### Attitudes regarding dengue fever

The average score of attitude regarding DF significantly differed among regencies (*P* < 0.001). The highest mean score of the attitude was obtained in Aceh Timur and the lowest in Langsa, 63.6 and 28.1, respectively (Table 1). Although more than 45% of the participants had good knowledge, only 32.1% (196 participants) had a good attitude regarding DF, and this was associated with education, occupation, SES and a personal history of DF (Table 3). As expected, having personally experienced DF was associated with approximately two times greater odds of having good attitude compared to participants who had not. In the final model, none of explanatory variable was associated with attitude towards DF (Table 3).

**Table 3:** Univariate and multiple logistic regression analysis showing predictors of attitude levels (good vs. poor) (*n* = 609)

Independent variable	Univariate		Multivariate	
	OR (95% CI)	*P*–value	aOR (95% CI)	*P*–value
Regency		< 0.001	–	–
Aceh Besar	1			
Aceh Tengah	0.30 (0.14–0.64)			
Langsa	0.00 (0.00–3.56)			
Aceh Utara	1.19 (0.20–2.31)			
Aceh Singkil	0.92 (0.47–1.81)			
Sabang	1.04 (0.54–2.02)			
Aceh Timur	1.78 (0.92–3.46)			
Aceh Selatan	0.30 (0.15–0.61)			
Aceh Tamiang	0.73 (0.34–1.35)			
Age group (years)		0.209		0.406
17–29	1		1	
30–44	1.02 (0.70–1.48)		1.32 (0.80–2.17)	
45–59	0.59 (0.23–1.11)		0.87 (0.40–1.89)	
60–84	0.24 (0.03–2.01)		0.45 (0.05–3.99)	
Sex		0.169		0.249
Male	1		1	
Female	1.30 (0.89–1.91)		1.27 (0.84–1.91)	
Education		< 0.001		0.035
Illiterate	1		1	
Primary school	0.50 (0.08–2.86)		0.42 (0.07–2.50)	
Junior high school	0.51 (0.10–2.65)		0.38 (0.07–2.05)	
Senior high school	1.58 (0.43–5.78)		1.16 (0.28–4.74)	
Diploma	2.20 (0.59–8.18)		1.62 (0.37–6.93)	
Graduated	3.00 (0.81–11.00)		2.06 (0.48–8.69)	
Occupation		0.025		0.953
Farmer	1		1	
Civil servant	2.67 (1.42–5.02)		1.12 (0.59–2.12)	
Private employee	2.63 (1.32–5.55)		1.06 (0.58–1.92)	
Entrepreneur	1.80 (0.92–3.52)		0.97 (0.42–2.22)	
Student/University student	2.28 (1.20–4.33)		1.29 (0.63–2.62)	
Religion		0.258	–	–
Muslim	1			
Other	0.48 (0.03–2.43)			
Marital status		0.658	–	–
Unmarried	1			
Married	0.86 (0.61–1.22)			
Widowed	0.74 (0.25–2.15)			
Monthly income (IDR)		0.382	–	–
< 1 million	1			
1 – ≤ 2 million	1.05 (0.67–1.65)			
2 – ≤ 3 million	1.09 (0.67–1.78)			
> 3 million	1.58 (0.94–2.65)			
Socioeconomic status		0.013		0.212
Poorest quintile	1		1	
2nd	1.14 (0.63–2.03)		0.80 (0.43–1.50)	
3rd	1.48 (0.84–2.61)		1.23 (0.67–2.24)	
4th	1.89 (1.08–3.31)		1.39 (0.75–2.60)	
Richest quintile	2.33 (1.34–4.05)		1.50 (0.80–2.83)	
Type of residence		0.050		0.943
City	1		1	
Suburb	0.69 (0.48–1.00)		0.98 (0.65–1.48)	
Family member(s) suffered from dengue fever		0.172		0.827
Yes	1		1	
No	0.75 (0.50–1.12)		1.05 (0.65–1.71)	
Personally experienced dengue fever		0.018		0.055
Yes	1		1	
No	0.51 (0.29–0.89)		0.52 (0.26–1.01)	

*aOR* adjusted odds ratio, *IDR* Indonesian rupiah

### Dengue fever prevention practices

In this study, 32.0% (195) of the participants had good DF prevention practices, a proportion similar to that of participants who had good attitude regarding DF. Factors correlated with prevention practice were education, occupation, SES and type of residence (Table 4). Participants who had a diploma degree or graduated from university were nine times more likely to have good DF prevention practice compared to those who were illiterate. In addition, participants who worked as civil servants, were employed in the private sector, were entrepreneurs or students also had higher odds of having good practice compared to farmers. Interestingly, there was no association between monthly income and preventive practice, although SES was significantly associated with prevention practice in a dose-dependent manner. Compared to the poorest SES, the odds of having a good DF prevention practices increased from 1.16 times for the second SES quintile, 1.41 times for the third SES quintile, 1.38 times for the fourth SES quintile to 2.68 times for the richest SES quintile (Table 4). In addition, as expected, participants living in the cities had better preventive practice compared to their counterparts in the suburbs (Table 4). In the multivariate analysis, increased odds of having good DF prevention practice was identified among participants from the 5th quintile compared to the poorest group (1st quintile) with OR: 2.68 (95% CI: 1.40–5.12) (Table 4).

**Table 4:** Univariate and multiple logistic regression analysis showing predictors of practice levels (good vs. poor) (*n* = 609)

Independent variable	Univariate		Multivariate	
	OR (95% CI)	*P-*value	aOR (95% CI)	*P*–value
Regency		0.017	–	–
Aceh Besar	1			
Aceh Tengah	0.42 (0.19–0.92)			
Langsa	0.49 (0.23–1.01)			
Aceh Utara	1.13 (0.56–2.25)			
Aceh Singkil	1.22 (0.61–2.46)			
Sabang	1.60 (0.81–3.16)			
Aceh Timur	1.40 (0.71–2.76)			
Aceh Selatan	1.09 (0.57–2.05)			
Aceh Tamiang	1.13 (0.56–2.25)			
Age group (years)		0.307		
17–29	1			
30–44	1.01 (0.69–1.47)			
45–59	0.74 (0.40–1.35)			
60–84	2.63 (0.69–9.99)			
Sex		0.131		0.057
Male	1		1	
Female	1.34 (0.91–1.96)		1.48 (0.99–2.24)	
Education		0.001		0.256
Illiterate	1		1	
Primary school	2.43 (0.24–24.03)		2.45 (0.23–25.25)	
Junior high school	1.80 (0.18–17.66)		1.44 (0.14–14.86)	
Senior high school	5.65 (0.72–43.90)		4.78 (0.57–39.81)	
Diploma	9.00 (1.15–70.42)		3.98 (0.46–33.88)	
Graduated	9.11 (1.17–70.88)		4.33 (0.51–36.67)	
Occupation		< 0.001		0.007
Farmer	1		1	
Civil servant	3.56 (1.78–7.10)		1.83 (1.05–3.17)	
Private employee	6.25 (3.00–12.98)		1.02 (0.59–1.78)	
Entrepreneur	2.97 (1.44–6.10)		0.51 (0.21–1.21)	
Student/University student	2.27 (1.11–4.61)		0.58 (0.32–1.05)	
Religion		0.261	–	–
Muslim	1			
Other	0.30 (0.03–2.45)			
Marital status		0.714	–	–
Unmarried	1			
Married	0.88 (0.62–1.24)			
Widowed	0.75 (0.26–2.18)			
Monthly income (IDR)		0.474	–	–
< 1 million	1			
1 – ≤ 2 million	1.01 (0.64–1.59)			
2 – ≤ 3 million	1.14 (0.70–1.86)			
> 3 million	1.49 (0.89–2.25)			
Socioeconomic status		< 0.001		0.015
Poorest quintile	1		1	
2nd	1.64 (0.90–2.97)		1.16 (0.61–2.19)	
3rd	1.82 (1.01–3.28)		1.41 (0.76–2.63)	
4th	1.94 (1.08–3.49)		1.38 (0.72–2.63)	
Richest quintile	3.88 (2.19–6.88)		2.68 (1.40–5.12)	
Type of residence		0.043		0.948
City	1		1	
Suburb	0.69 (0.48–0.98)		1.01 (0.67–1.53)	
Family member(s) suffered from dengue fever		0.228		0.768
Yes	1		1	
No	0.78 (0.52–1.16)		0.92 (0.56–1.51)	
Personally experienced dengue fever		0.223		0.442
Yes	1		1	
No	0.70 (0.40–1.23)		0.76 (0.38–1.51)	

*aOR* adjusted odds ratio, *IDR* Indonesian rupiah

### Correlation between knowledge, attitude, practice and socioeconomic status

There was a significant positive correlation between asset score (socioeconomic status) and KAP scores (Table 5). The significant correlations between KAP scores and asset score indicated that knowledge, attitude and practice regarding DF increased with increasing SES.

**Table 5:** Correlation between score of knowledge, attitude, practice and asset score (socioeconomic status)

Variables	Correlation (95% CI)	*P-*value
Asset index-knowledge	0.27 (0.21–0.33)	< 0.001
Asset index-attitude	0.16 (0.09–0.23)	< 0.001
Asset index-practice	0.26 (0.19–0.32)	< 0.001
Knowledge-attitude	0.34 (0.28–0.40)	< 0.001
Knowledge-practice	0.21 (0.15–0.27)	< 0.001
Attitude-practice	0.27 (0.21–0.33)	< 0.001

There was also a significant positive correlation between knowledge-attitude, knowledge-practice and attitude-practice with the strongest correlation identified for knowledge-attitude. To validate these results, further analysis was conducted using KAP scores that had been classified as “good” and “poor” based on a cut-off point of 80%. Our analysis showed that participants who had good knowledge were 2.5 times more likely to have a good attitude regarding DF (OR: 2.66, 95% CI: 1.87–3.77). However, there was no strong association between good knowledge and good practice (OR: 1.41; 95% CI: 1.00–1.98). As predicted, there was a strong association between good attitude and good DF preventive practice (OR: 2.18; 95% CI: 1.52–3.11).

### Sources of information on dengue fever

**Fig. 2:**
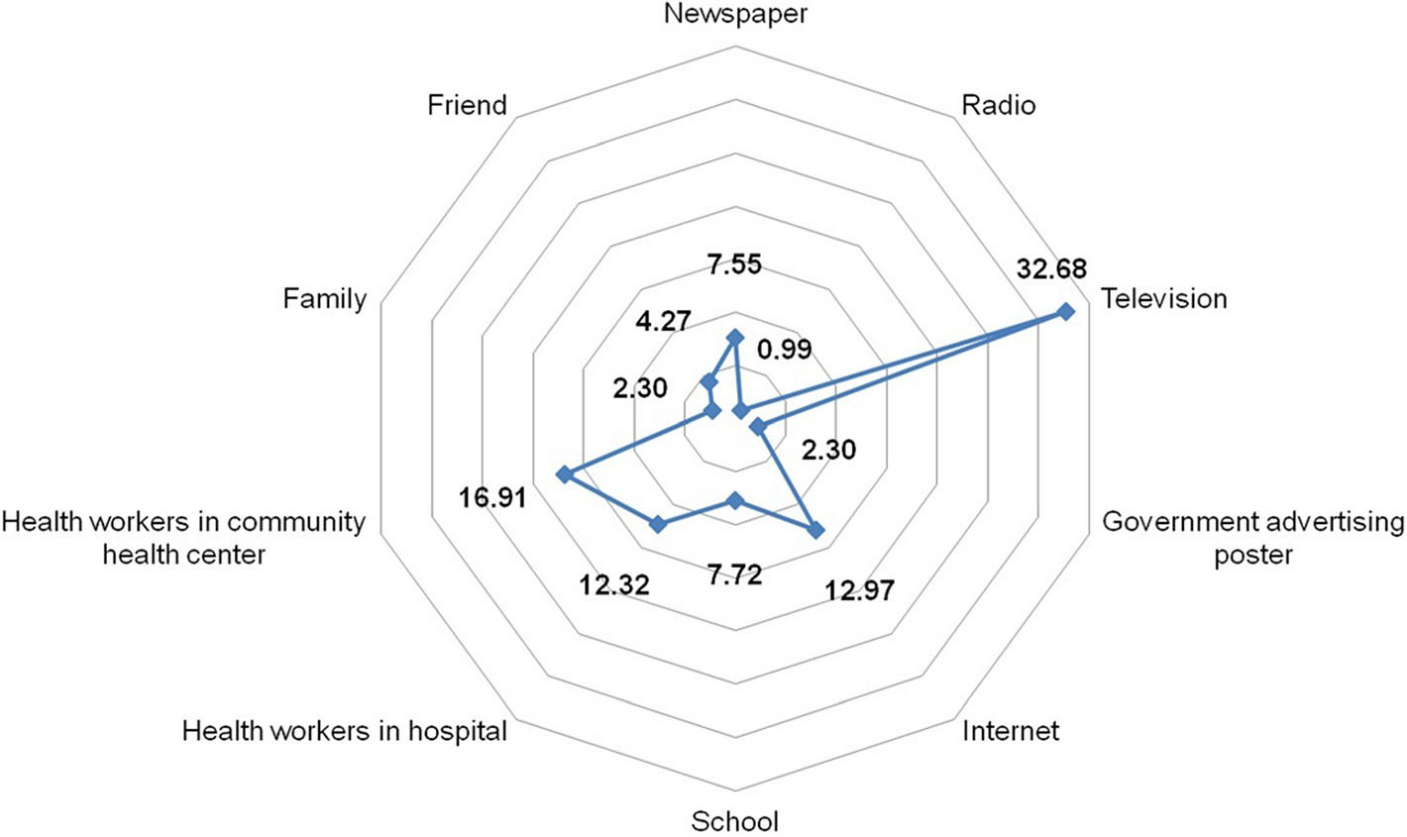
Source of information on dengue fever among participants

In this study, television (32.7%), health care workers (HCWs) in the Community Health Centre (Puskesmas) (16.9%), internet (13.0%) and HCWs in the hospital (12.3%) were the major sources of information on DF among participants (Fig. 2). Only 1% of the participants received information on DF from the radio. A possible reason is that few of the participants owned a radio. This indicates that radio transmission may not be an important source of information in Aceh.

Interestingly, there was a significant difference regarding the source of information on DF between participants who did, or did not, have family member(s) who had suffered from DF (*P* ≤ 0.001). Although the most prominent source was television in both groups (32.7% vs. 34.4%), 19.0% of the participants without any history of DF in their family had received information on DF from HCWs in Puskesmas compared to 12.6% of those whose family members had suffered from DF. In addition, internet was a source of information on DF among 19.3% of those participants whose family members had suffered from DF compared to only 11.2% in the group without family history of DF. Among participants who had personally experienced DF, more than 20% received information on DF from the internet and 7.1% from HCWs in Puskesmas. In contrast, 17.9% of the participants who had never suffered from DF received this information from HCWs in Puskesmas and only a tenth of them obtained it from the internet.

## Discussion

This study provides the first description of KAP regarding DENV transmission and its prevention in Aceh, Indonesia, where an upward trend in DF incidence has been recorded in the aftermath of the 2004 earthquake and tsunami. These findings may contribute to the identification of intervention groups for DF prevention programs and to the design and development of intervention programs to protect the health of vulnerable groups in the community.

This study found more than 50% of the participants to have poor knowledge regarding DF. The lowest average scores of knowledge regarding DF were obtained in Aceh Tengah, a regency located in the central region of Aceh at approximately 1200 m above sea level. The regency experiences the coolest temperatures in Aceh and has a very low DF incidence due to the low number of competent vectors for DF. In 2012 there were no recorded fatalities due to DF in Aceh Tengah. In contrast, the regency of Aceh Timur (mainly lowland) was among those with the highest knowledge scores; the DF case fatality rate in this regency was 5.9% in 2012 [21], indicating that knowledge regarding DF is closely related to the occurrence and mortality of DF. This finding is consistent with those of a previous study in Nepal showing that the knowledge regarding DF was lower among highland community members, as consequence of lower exposure to the vectors and the diseases, when compared to lowland communities [11].

In univariate analysis, factors associated with good knowledge regarding DF were higher levels of education, occupation (civil servants, private sector employees, entrepreneurs, students), unmarried status, high monthly family income, high SES, and living in the city. There was a robust association between formal education and knowledge regarding DF in the present study. For example, a person with a diploma was almost seven times more likely to have good knowledge compared to a person who was illiterate. This association increased significantly, to almost 14 times more likely, among persons who graduated from a university with a degree. One of the reasons for this may be that school and university curricula of different countries vary in their content on DF which in turn may affect the knowledge level among literate people. In addition, a strong association between income and knowledge level was found in the univariate analysis. However, our multivariate regression analysis revealed that monthly income and other factors (education and occupation) were confounding factors for the SES, and SES was the only strong independent predictor for the knowledge domain regarding DF. One of the possible reasons for the association between SES and better knowledge regarding DF is that people with higher economic status might have better access to information sources on DF [22]. Castro et al. [22] postulated that the combination of better access to information about DF and higher education level might assure a better understanding and comprehension of information on DF when accessed; therefore, better knowledge regarding DF could be achieved.

We found a weak association between knowledge of DF and preventive practice. Although more than 45% of the participants had good knowledge regarding DF (based on the 80% cut-off point), only 32% had good preventive practice. For example, 94% of the participants understood that windows screens and bed nets reduce mosquito biting, but only 74% actually used window screens. In addition, although 91% of the participants understood that DENV vectors breed in standing water, only 73% changed the water in flower containers regularly. Less than half of the participants with good knowledge regarding DF had good preventive practice. Our results indicate that translation from knowledge to practice among participants was poor, as has been reported elsewhere [11, 23–25]. Although the exact factor that inhibits the translation from knowledge regarding DF into preventive practice is unknown, we suspect that SES could be one of the major factors among the participants of our study. This is based on the following rationale: Firstly, our analysis found that low education and working as a farmer were associated with low preventive practice. As mentioned before, education and occupation were confounding factors for SES. Secondly, living in the suburbs was also associated with low preventive practice, and the majority of residents in the suburbs had a lower education level and were poorer (their main occupation being farming) compared to their counterparts in the cities. We see a major problem in the time allocation for farmers, who have to work every day whereas civil servants or those working in private sector employment have a holiday within the weekend. Less time available could be the main reason for less knowledge regarding DF among suburban participants, and this reason could be a major obstacle for the translation of knowledge into preventive practice among people with low SES. Persons who can spare time during weekends might have used part of that time for translating their knowledge of DF into preventive practices.

As predicted, this study found a strong association between good attitude regarding DF and good practice, indicating that the translation of attitudes into practice was good. Therefore, appropriate preventive programs should be designed to increase not only the knowledge of people but also to improve their attitude regarding DF.

To tackle low knowledge among inhabitants of the suburbs and a lack of translation of knowledge into practice at the same time, the role of religion in the community should be better appreciated. Approximately 98% of the inhabitants of Aceh are Muslim and a male adult is compulsory to pray five times a day especially every Friday afternoon in the mosque. This occasion could be used in close cooperation with mosque management staff to spread educational materials on DF and its prevention, such as brochures and fact sheets. In addition, the preachers should be encouraged to include health and environmental topics, including DF, in the materials used during speech session after praying. To expand this, mosque management could include healthcare providers to provide DF-related speeches in formal or non-formal religious lecture sessions. Furthermore, to increase DF prevention practices, mosque management staff could request the community members to do voluntary communal works to clean and bury of water containers around dwellings, clear the neighborhood of ponds and pits and clean the gutters and surface water drains, especially before and during raining season. Up to now, such opportunities have never been used in Aceh. Engaging local religious leaders and mosques would have at least two advantages: First, it could be more convenient (in terms of time spent) for people to receive information at or near the mosque compared to seminars conducted either in Puskesmas or the town hall (where attendance is usually very low). Second, most of the Acehnese are very observant Muslims and more likely to follow their religious leader in the mosque compared to HCWs. Therefore, it might be easier and more effective to disseminate information on DF and its prevention in this setting. In addition, Aceh is the only province in Indonesia that is implementing Islamic Sharia Law and therefore the religion-related approach will be easier to be implemented in Aceh context. However, it should be kept in mind that people’s attention to such topics is not very high when there is no DF case in the community.

In order to specifically improve the translation of knowledge and attitude into real preventive practice, the most appropriate target groups are hospitalized DF patients and their family members visiting the hospital. The rationale behind this is: (i) we found that the knowledge regarding DF of these groups was not significantly increased compared to participants who had never suffered from DF and had no family history of DF, indicating that the existing DF prevention program did not educate them enough; (ii) we also found that these groups had better attitude regarding DF indicating higher awareness; and (iii) patients’ family members could be more easily interested due to their perceived susceptibility of contracting DF because they are living together with the patient. Increased perceived susceptibility is an important driver of DF prevention practices [26].

In our study context, the status of previous dengue infection of participants (i.e the presence of the antibody anti-dengue) is not relevant. As a country with hyperendemic for dengue, almost 90% of the inhabitants aged 15–18-year-olds in Indonesia are seropositive for dengue and most of the inhabitants with seropositive are never symptomatic [27]. What we are interested is the inhabitants who experienced symptomatic dengue previously (i.e experienced symptomatic dengue). This history is important because, in nature, such experience will associate with awareness and attitude towards disease. In this study, we did record the information of symptomatic dengue history in respondents. We observed a higher use of the internet as a source of information on DF among participants who had experienced DF personally or in their family. This indicates that they might have tried to get more information regarding DF from the internet during and/or after the DF episode reflection awareness of the disease. Therefore, both of these groups (patients and their families) appear most suitable for DF education during visits to Puskesmas or hospitals*.* Dengue fever education materials could be delivered directly by HCWs to the patients or by providing family members with posters, brochures, or booklets while in hospital. This strategy could be particularly effective in Aceh where a cultural practice exists in which patients in hospital are visited not only by members of the core family but also by the extended family and neighbors. All of these should be educated on such opportunities, improving their knowledge as well as attitude and preventive practice regarding DF. In addition, they should be tasked with spreading DF education materials among their neighbors (e.g., one person should spread the education materials to at least five relatives or neighbors). By adopting a “one for five” strategy, dissemination of the information could be effective. Beyond the mere dissemination of information materials on DF, the “one for five” strategy can also deliver “blue messages” from persons who visited the DF patient in hospital. As Acehnese people tend to explain the condition of their family members in hyperbolic phrases, it is very likely that this could drive the awareness of interlocutors. However, further study should be conducted to assess the effectiveness of such a “one for five” strategy in increasing the KAP regarding DF in Aceh.

As expected, this study found a negative association between receiving information from HCWs in Puskesmas and personal history of DF. In Indonesia, the main role of Puskesmas is to implement preventive programs while the major role of hospitals is curative. Therefore, Puskesmas might be one of the most suitable places for implementing a DF prevention program in order to improve preventive practices among local residents. To achieve this in the future, the government should encourage HCWs in Puskesmas to better educate DF patients and their family members and neighbors, also applying community outreach methods like the “one for five” strategy.

Inevitably, there are some limitations of the present study. First, this study could not determine how all the reported practices were translated into actual practice because the interviewers did not directly inspect the houses inhabited by participants. Second, a desirability bias might exist in some questions within the attitude domain. This latter issue has also been reported from similar study in Nepal [11].

## Conclusions

In Aceh, Indonesia, the knowledge regarding DF is low among inhabitants. Only one-third of the participants had good attitude towards DF and reported good preventive practices. Although SES was the only independent predictor factor for KAP domains in this study, some of the intervention groups that should be considered for a DF prevention program are inhabitants with low SES, inhabitants with low education level, those living in the suburbs, and farmers. There was a strong association between knowledge and attitude regarding DF, and between attitude and preventive practice. However, there was a poor translation of knowledge into preventive practice. To achieve success in DF prevention, programs should be designed to increase not only knowledge and attitude domains but also the translation of these domains into real preventive measures. To disseminate DF information and increase the translation of knowledge into preventive measures, a religion-based approach might be considered as part of preventive programs in Aceh. In this study we found that having a personal or family history of DF was not associated with an increased knowledge regarding DF. The most likely explanation for this worrying result could be insufficient information on DF given by HCWs during the treatment of patients in Puskesmas or hospital. In addition, we found a negative association between personal history of DF and receiving information from HCW in Puskesmas. To address these problems, the critical role of Puskesmas as the frontline facility in disease prevention should be optimized using two strategies. First, HCWs should be empowered and encouraged to better educate DF patients, their families and neighbors during their visits to the Puskesmas. Second, Puskesmas and hospital should provide DF patients, their families and visiting neighbors with adequate health education materials, applying outreach strategies that use these groups of persons as multipliers in their communities. In addition, to disseminate DF information to the broader community, posters, booklets and brochures must also be distributed to schools, universities and other various public administrative offices. To enhance the awareness, simple and educating DF posters could be posted in public areas that everyone from different educational levels can understand.

## Additional files

Additional file 1: Completed of STROBE checklist of the study. (PDF 236 kb)

Additional file 2: Distribution of the knowledge regarding dengue fever among participant groups with different socioeconomic level. (PDF 154 kb)

Additional file 3: Distribution of attitude regarding dengue fever among participant groups with different socioeconomic level. (PDF 195 kb)

Additional file 4: Distribution of good practice regarding dengue fever prevention among participant groups with different socioeconomic level. (PDF 101 kb)

## Abbreviations

aOR: Adjusted odds ratio
CI: Confident interval
DENV: Dengue virus
DF: Dengue fever
HCW: Health care worker
KAP: Knowledge, attitude, and practice
OR: Odds ratio
Puskesmas: Community Health Centre
SES: Socioeconomic status
