# Supplementary material for: Knowledge, attitude, and practice regarding dengue virus infection among inhabitants of Aceh, Indonesia: a cross-sectional study
Source: BMC Infect Dis. 2018 Feb 27;18:96. doi: 10.1186/s12879-018-3006-z (PMC5830327; doi:10.1186/s12879-018-3006-z)
Supplement: Supplementary file 1 — Completed of STROBE checklist of the study. (PDF 236 kb) [file 12879_2018_3006_MOESM1_ESM.pdf]

STROBE Statement - Checklist of items that should be included in reports of cross-sectional studies

Items	Item No	Recommendation	Subheading of article
**Title and abstract**	1	(a) Indicate the study's design with a commonly used term in the title or the abstract<br>*Knowledge, attitude, and practice regarding dengue virus infection among inhabitants of Aceh, Indonesia: A cross-sectional study*	*Title*
		(b) Provide in the abstract an informative and balanced summary of what was done and what was found<br>*Abstract in this study consisting of background, method, result and conclusion sections with informative and balanced information.*	*Abstract*
**Introduction**			
Background/rationale	2	Explain the scientific background and rationale for the investigation being reported<br>*We provided specific background related to the important of dengue fever (DF) in public health, increasing number of dengue fever in Indonesia and Aceh and the failed of dengue preventive program in Aceh. We stated in the end of Background section: "Despite the increasing incidence of dengue fever in Aceh there has been no study to assess the knowledge, attitude and practice (KAP) of Aceh communities regarding dengue virus (DENV) transmission and its prevention."*	*Background*
Objectives	3	State specific objectives, including any prespecified hypotheses (N/A)<br>*"This study aimed to assess and compare the KAP among community groups in Aceh, in order to design intervention strategies for an effective dengue prevention program."*	*Background*
**Methods**			
Study design	4	Present key elements of study design early in the paper<br>*This study was cross-sectional study. "A cross-sectional study was conducted in the province of Aceh, which is located in the westernmost region of the Indonesian archipelago and has a surface area of 57,956 km^2."*	*Study design and setting*
Setting	5	Describe the setting, locations, and relevant dates, including periods of recruitment (N/A), exposure (N/A), follow-up (N/A), and data collection<br>*Setting of study: Healthy inhabitants that meet inclusion criteria were interview after informed concerned. Locations of study: "This study was conducted in province of Aceh included localities in the southwestern, central and northern regions (in seven regencies (Aceh Tengah, Aceh Besar, Aceh Utara, Aceh Singkil, Aceh Timur, Aceh Selatan and Aceh Tamiang) and two municipalities (Langsa and Sabang)). Relevant dates of study or data collection: "Data were collected from November 2014 to March 2015."*	*Study design and setting & Interview and data collection.*
Participants	6	(a) Give the eligibility criteria, and the sources and methods of selection of participants<br>*Eligible criteria in this study: "All inhabitants who were aged over 16 years, had resided in the specified regency or municipality for more than 3 months, and were able to communicate were considered to be eligible for inclusion as participants of the study." Sources and methods of selection of participants: "So far no data related to the KAP towards dengue in Aceh have been available. Therefore, to calculate a representative sample size for the Aceh population (4,791,924), we assumed that 50% of participants would have good KAP regarding dengue. With a 5% margin of error and 95% confidence level, 385 participants were required to achieve the minimum recommended sample size."*	*Sampling and sample size*

Items	Item No	Recommendation	Subheading of article
Variables	7	Clearly define all outcomes, exposures (N/A), predictors, potential confounders, and effect modifiers (N/A). Give diagnostic criteria (N/A)<br>*Response variables or outcome of the study: Knowledge regarding DF, attitude towards DF, and preventive measures against dengue virus. Explanatory variables or predictors in this study: age, education, occupation, religion, marital status, income, and type of residence of participants, and whether or not they or family members had already suffered from DF, and socioeconomic status. "Confounding factors were explored by comparing the difference between the adjusted odds ratio (aOR) in multivariate analyses and the crude odds ratio (OR) in univariate analyses, of a particular predictor variable on the KAP domain."*	*Explanatory variables, Response variables & Statistical analysis*
Data sources/ measurement	8*	For each variable of interest, give sources of data and details of methods of assessment (measurement). Describe comparability of assessment methods if there is more than one group.<br>*Interest variables (included explanatory and response variables) were assessed by interviews. "To facilitate the interviews, a set of validated and pre-tested questionnaires, covering explanatory and response variables was used. Interviews were conducted in Bahasa Indonesia by data collection team." In this study, all respondents were healthy individuals and the same method was used to assess variables of interest in all respondents.*	*Study instrument & Interview and data collection*
Bias	9	Describe any efforts to address potential sources of bias<br>*During interview, "to avoid the bias, the correct answers to the survey questions were not provided to interviewers." In addition, "during analysis, confounding factors were explored by comparing the difference between the adjusted odds ratio (aOR) in multivariate analyses and the crude odds ratio (OR) in univariate analyses, of a particular predictor variable on the KAP domain."*	*Interview and data collection & Analysis*
Study size	10	Explain how the study size was arrived at<br>*In this study, study size refers to sample size. The information regarding sample size is given in Participants (Item 6).*	*Sampling and sample size*
Quantitative variables	11	Explain how quantitative variables were handled in the analyses. If applicable, describe which groupings were chosen and why<br>*All explanatory variables were divided into group to give quantitative measures. In addition, socioeconomic statuses were divided in to five categories 1st to 5th quintile based on the asset index calculation. One category of each variable was used as reference category (usually group that was assumed associated with poor response variable). Response variables (KAP) were dichotomized into good and poor group based on 80% cut-off point. These processes resulted all variables become quantitative and therefore suitable for further analyses.*	*Explanatory variables, Response variables & Statistical analysis*
Statistical methods	12	(*a*) Describe all statistical methods, including those used to control for confounding<br>*"To determine the role of socio-demographic characteristics on KAP on dengue fever, differences in socio-demographic status were compared with the KAP levels (good and poor) using the Chi Square-Test, ANOVA or Fisher's exact test as appropriate. Logistic regression analysis was used to determine the predictors of each KAP domain." In addition, "confounding factors were explored by comparing the difference between the adjusted odds ratio (aOR) in multivariate analyses and the crude odds ratio (OR) in univariate analyses, of a particular predictor variable on the KAP domain."*	*Statistical analysis*
		(*b*) Describe any methods used to examine subgroups and interactions<br>*In this study, correlation between response variables (KAP) were assessed Spearman's rank correlation (r_s). "This correlation was chosen because the KAP scores were not normally distributed as revealed by the Kolmogorov–Smirnov test."*	*Statistical analysis*

Items	Item No	Recommendation	Subheading of article
		(*c*) Explain how missing data were addressed<br>*In this study, we only included data of participants who provided or completed all section of the questionnaire. All participants with missing data were excluded from analyses.*	*Study population characteristics*
		(*d*) If applicable, describe analytical methods taking account of sampling strategy<br>*There is no problem related sampling strategy in our study, but the analytical analysis in this study was choose based on distribution of our data. For example in the univariate analysis we used Chi Square-Test, ANOVA or Fisher's exact test as appropriate normality of the data. Also, we used Spearman's rank correlation (r_s) in correlation analysis based on normality test of the data using Kolmogorov–Smirnov test.*	*Statistical analysis*
		(*e*) Describe any sensitivity analyses<br>*There is no any sensitivity analysis relevant to this study. However, we did questionnaire validity test to assess the internal consistency of the questionnaire prior used in the study.*	*Questionnaire validity test*
**Results**			
Participants	13*	(a) Report numbers of individuals at each stage of study—eg numbers potentially eligible, examined for eligibility, confirmed eligible, included in the study, completing follow-up (N/A), and analysed.<br>*In this study, "677 healthy community members were surveyed and 68 (10.0%) participants were excluded from the analysis due to missing information. A total of 609 inhabitants, who provided data for all sections of questionnaire, were included in the final analysis (Table 1)." Unfortunately, in this study, the number of potentially eligible inhabitants was not recorded.*	*Study population characteristics*
		(b) Give reasons for non-participation at each stage<br>*In this study, the non-participant occurred in one stage only which was incomplete data during data collection. All incomplete data from participants were excluded from the analysis.*	*Study population characteristics*
		(c) Consider use of a flow diagram	
Descriptive data	14*	(a) Give characteristics of study participants (eg demographic, clinical, social) and information on exposures and potential confounders<br>*In this study, characteristics of study participants are summarized in Table 1. We included a very little information of the Table 1 into description text to avoid repetitive.*	*Study population characteristics*
		(b) Indicate number of participants with missing data for each variable of interest<br>*In this study, we only included data of participants who provided or completed all section of the questionnaire. Meaning that each variable of interest had the same number of participants.*	*Study population characteristics*
Outcome data	15*	Report numbers of outcome events or summary measures<br>*The number of each categories of the knowledge (good vs. poor), attitude (good vs. poor), and practice (good vs. poor) regarding DF are given in the three different sub-heading: Knowledge about signs and symptoms of dengue fever and transmission of dengue virus, and Attitudes regarding dengue fever Dengue fever prevention practices. The number of participants for each level of socioeconomic status was also provided in the Result section.*	*Knowledge about signs and symptoms of dengue fever and transmission of dengue virus, Attitudes regarding dengue fever & Dengue fever prevention practices.*
Main results	16	(*a*) Give unadjusted estimates and, if applicable, confounder-adjusted estimates and their precision (eg, 95% confidence interval). Make clear which confounders were adjusted for and why they were included.	*Table 2, Table 3 & Table 4. Discussion*

Items	Item No	Recommendation	Subheading of article
		In this study, unadjusted estimates (univariate analysis) and adjusted estimates are calculated for each explanatory and response variable and both of them provided in Table 2,3 and 4 for knowledge, attitude and practice, respectively. Confounding factors were assessed and discussed in the Discussion section.	
		(*b*) Report category boundaries when continuous variables were categorized<br>*In this study, KAP variable were categorized into "good" or "poor" based on an 80% cut-off point. In addition, continuous variable of asset index (socioeconomic status) also classified into 1st to 5th quintile wherein the 1st quintile is the poorest and the 5th the least poor. These category criteria used throughout the manuscript.*	*Statistical analysis, Knowledge about signs and symptoms of dengue fever and transmission of dengue virus, Attitudes regarding dengue fever & Dengue fever prevention practices.*
		(*c*) If relevant, consider translating estimates of relative risk into absolute risk for a meaningful time period	
Other analyses	17	Report other analyses done—eg analyses of subgroups and interactions, and sensitivity analyses<br>*In this study, correlation between variables of response variables (KAP) and association between response variables (KAP) and socioeconomic status were also assessed.*	*Correlation between knowledge, attitude, practice and socioeconomic status*
**Discussion**			
Key results	18	Summarise key results with reference to study objectives<br>*The key findings are explained throughout the discussion section with comparison with other studies.*	*Discussion*
Limitations	19	Discuss limitations of the study, taking into account sources of potential bias or imprecision. Discuss both direction and magnitude of any potential bias.<br>*Here we discussed at least two limitation of our study. "First, this study could not determine how all the reported practices were translated into actual practice because the interviewers did not directly inspect the houses inhabited by participants. Second, a desirability bias might exist in some questions within the attitude domain"*	*Discussion*
Interpretation	20	Give a cautious overall interpretation of results considering objectives, limitations, multiplicity of analyses, results from similar studies, and other relevant evidence.<br>*Some cautious are given in the discussion related to our finding and our proposed approaches in dengue prevention program. Such as: "However, it should be kept in mind that people's attention to such topics is not very high when there is no DF case in the community". "However, further study should be conducted to assess the effectiveness of such a "one for five" strategy in increasing the KAP regarding DF in Aceh". Then we also mentioned the limitation of our study in the end of discussion.*	*Discussion*
Generalisability	21	Discuss the generalisability (external validity) of the study results.<br>*Some generalisabilities of the results from this study were discussed especially in the larger context such as Aceh and Indonesia in general.*	*Discussion*
**Other information**			
Funding	22	Give the source of funding and the role of the funders for the present study and, if applicable, for the original study on which the present article is based.	*Funding*

Items	Item No	Recommendation	Subheading of article
		Funding: Not applicable.	

*Give information separately for exposed and unexposed groups.

Note: An Explanation and Elaboration article discusses each checklist item and gives methodological background and published examples of transparent reporting. The STROBE checklist is best used in conjunction with this article (freely available on the Web sites of PLoS Medicine at http://www.plosmedicine.org/, Annals of Internal Medicine at http://www.annals.org/, and Epidemiology at http://www.epidem.com/). Information on the STROBE Initiative is available at www.strobe-statement.org.
