# Supplementary material for: Knowledge, attitude, and practice regarding dengue virus infection among inhabitants of Aceh, Indonesia: a cross-sectional study
Source: BMC Infect Dis. 2018 Feb 27;18:96. doi: 10.1186/s12879-018-3006-z (PMC5830327; doi:10.1186/s12879-018-3006-z)
Supplement: Supplementary file 2 — Distribution of the knowledge regarding dengue fever among participant groups with different socioeconomic level. (PDF 154 kb) [file 12879_2018_3006_MOESM2_ESM.pdf]

Additional file 2 – Table. Distribution of the knowledge regarding dengue fever among participant groups with different socioeconomic level

Variables	1st quintile (n=122) n (%)	2nd quintile (n=123) n (%)	3rd quintile (n=122) n (%)	4th quintile (n=121) n (%)	5th quintile (n=121) n (%)	*P*-value*
Dengue is caused by a virus. Yes	95 (77.9)	109 (88.6)	87 (71.3)	102 (84.3)	106 (87.6)	0.002
Can all mosquitoes transmit dengue virus? No	72 (59.0)	91 (74.0)	82 (67.2)	93 (76.9)	101 (83.5)	0.000
Do the *Aedes* mosquitoes transmit dengue virus? Yes	106 (86.9)	111 (90.2)	115 (94.3)	114 (94.2)	115 (95.0)	0.087
Do flies transmit dengue virus? No	75 (61.5)	99 (80.5)	103 (84.4)	97 (80.2)	101 (83.5)	0.000
Do ticks transmit dengue virus? No	82 (67.2)	99 (80.5)	98 (80.3)	98 (81.0)	100 (82.6)	0.023
Does casual person to person contact transmit DF? No	62 (50.8)	84 (68.3)	71 (58.2)	76 (62.8)	80 (66.1)	0.052
Is dengue virus transmitted through food and water? No	81 (66.4)	91 (74.0)	94 (77.0)	93 (76.9)	95 (78.5)	0.196
Do mosquitoes breed in standing water? Yes	106 (86.9)	113 (91.9)	113 (92.6)	110 (90.9)	115 (95.0)	0.236
Do window screens and bed net reduce mosquitoes? Yes	108 (88.5)	115 (93.5)	114 (93.4)	115 (95.0)	119 (98.3)	0.034
Do insecticide sprays (such as Baygon) reduce mosquitoes and prevent DF? Yes	103 (84.4)	112 (91.1)	113 (92.6)	116 (95.9)	117 (96.7)	0.003
Do tightly covered water containers reduce mosquitoes? Yes	110 (90.2)	115 (93.5)	114 (93.4)	113 (93.4)	117 (96.7)	0.377
Do mosquito repellents prevent mosquito bites? Yes	110 (90.2)	108 (87.8)	111 (91.0)	114 (94.2)	117 (96.7)	0.090
Is dengue virus transmitted to humans by the bite of female *Aedes* mosquitoes that have been infected? Yes	62 (50.8)	61 (49.6)	54 (44.3)	68 (56.2)	82 (67.8)	0.004
Is the rainy season the only season when DF is present? No	58 (47.5)	76 (61.8)	76 (62.3)	73 (60.3)	68 (56.2)	0.111
Can you identify *Aedes* mosquitoes? Yes	36 (29.5)	47 (38.2)	45 (36.9)	59 (48.8)	63 (52.1)	0.002
When are the *Aedes* mosquitoes most likely to feed/bite? Day time	24 (19.7)	51 (41.5)	60 (49.2)	55 (45.5)	56 (46.3)	0.000
Is headache a symptom of DF? Yes	103 (84.4)	96 (78.0)	95 (77.9)	95 (78.5)	113 (93.4)	0.005
Is joint pain a symptom of DF? Yes	100 (82.0)	103 (83.7)	100 (82.0)	107 (88.4)	114 (94.2)	0.024

Variables	1st quintile (n=122)<br><br>n (%)	2nd quintile (n=123)<br><br>n (%)	3rd quintile (n=122)<br><br>n (%)	4th quintile (n=121)<br><br>n (%)	5th quintile (n=121)<br><br>n (%)	*P*-value*
Is muscle pain a symptom of DF? Yes	90 (73.8)	99 (80.5)	87 (71.3)	97 (80.2)	111 (91.7)	0.001
Is pain behind the eyes a symptom of DF? Yes	66 (54.1)	71 (57.7)	70 (57.4)	69 (57.0)	93 (76.9)	0.002
Is bone sore a symptom of DF? Yes	77 (63.1)	90 (73.2)	93 (76.2)	92 (76.0)	100 (82.6)	0.012
Are nausea/vomiting symptoms of DF? Yes	93 (76.2)	99 (80.5)	95 (77.9)	94 (77.7)	97 (80.2)	0.919
Is rash a symptom of DF? Yes	113 (92.6)	118 (95.9)	119 (97.5))	115 (95.0)	119 (98.3)	0.183
Is diarrhea common in DF? Yes	61 (50.0)	53 (43.1)	50 (41.0)	51 (42.1)	64 (52.9)	0.242
Is stomach pain common in DF? Yes	59 (48.4)	58 (47.2)	52 (42.6)	53 (43.8)	69 (57.0)	0.181
Is gum bleeding common in DF? Yes	68 (55.7)	73 (58.3)	71 (58.2)	74 (61.2)	88 (72.7)	0.062
Can a person suffer from DF more than once? Yes	90 (73.8)	109 (88.6)	106 (86.9)	106 (87.6)	112 (92.6)	0.000
Does DF affect infants, children and adults? Yes	105 (86.1)	114 (92.7)	119 (97.5)	120 (99.20	119 (98.3)	0.000

*Analyzed by ANOVA, but Fisher`s exact test was used where more than 20% of the cells had expected cell counts less than five
