# Supplementary material for: Knowledge, attitude, and practice regarding dengue virus infection among inhabitants of Aceh, Indonesia: a cross-sectional study
Source: BMC Infect Dis. 2018 Feb 27;18:96. doi: 10.1186/s12879-018-3006-z (PMC5830327; doi:10.1186/s12879-018-3006-z)
Supplement: Supplementary file 3 — Distribution of attitude regarding dengue fever among participant groups with different socioeconomic level. (PDF 195 kb) [file 12879_2018_3006_MOESM3_ESM.pdf]

Additional file 3 – Table. Distribution of attitude regarding dengue fever among participant groups with different socioeconomic level

Variables	1st quintile (n=122)<br>n (%)	2nd quintile (n=123)<br>n (%)	3rd quintile (n=122)<br>n (%)	4th quintile (n=121)<br>n (%)	5th quintile (n=121)<br>n (%)	P-value*
Is DF a serious illness?						0.013
Strongly agree	57 (46.7)	59 (48.0)	43 (35.2)	53 (43.8)	64 (52.9)	
Agree	35 (28.7)	43 (35.0)	53 (43.4)	45 (37.2)	43 (35.5)	
Not sure	10 (8.2)	2 (1.6)	1 (0.8)	2 (1.6)	2 (1.7)	
Disagree	10 (8.2)	6 (4.9)	12 (9.8)	10 (8.3)	4 (3.3)	
Strongly disagree	10 (8.2)	13 (10.5)	13 (10.8)	11 (9.1)	8 (6.6)	
Are you at risk of getting DF?						0.325
Strongly agree	16 (13.1)	17 (13.8)	15 (12.3)	13 (10.7)	26 (21.5)	
Agree	43 (35.2)	39 (31.7)	41 (33.6)	43 (35.5)	49 (40.5)	
Not sure	16 (13.1)	23 (18.7)	18 (14.8)	24 (19.8)	13 (10.7)	
Disagree	36 (29.5)	34 (27.6)	33 (27.0)	33 27.3)	22 (18.2)	
Strongly disagree	11 (9.0)	10 (8.2)	15 (12.3)	8 (6.6)	11 (9.1)	
Can DF be prevented?						
Strongly agree	41 (33.6)	45 (36.6)	42 (34.4)	43 (35.5)	67 (55.4)	0.097
Agree	57 (46.7)	51 (41.5)	50 (41.0)	53 (43.8)	40 (33.1)	
Not sure	8 (6.6)	8 (6.5)	5 (4.1)	7 (5.8)	3 (2.4)	
Disagree	11 (9.0)	9 (7.3)	14 (11.5)	10 (8.3)	6 (5.0)	
Strongly disagree	5 (4.1)	10 (8.1)	11 (9.0)	8 (6.6)	5 (4.1)	
Is controlling the breeding places of mosquitoes a good strategy to prevent DF?						0.002
Strongly agree	30 (24.6)	48 (39.0)	35 (28.7)	47 (38.8)	53 (43.8)	
Agree	53 (43.4)	47 (38.2)	52 (42.6)	46 (38.0)	49 (40.5)	
Not sure	21 (17.2)	7 (5.7)	6 (4.9)	7 (5.8)	10 (8.3)	
Disagree	11 (9.0)	13 (10.6)	22 (18.0)	15 (12.4)	6 (5.0)	
Strongly disagree	7 (5.7)	8 (6.5)	7 (5.7)	6 (5.0)	3 (2.5)	
Do you think that stagnant water around the house in discarded tyres, broken pots and bottles is a breeding place of Aedes mosquitoes?						0.014
Strongly agree	37 (30.3)	52 (42.3)	43 (35.2)	52 (43.0)	67 (55.4)	
Agree	53 (43.4)	41 (33.3)	45 (36.9)	45 (37.2)	39 (32.2)	
Not sure	13 (10.7)	10 (8.1)	10 (8.2)	3 (2.5)	4 (3.3)	
Disagree	13 (10.7)	9 (7.3)	14 (11.5)	10 (8.3)	8 (6.6)	
Strongly disagree	6 (4.9)	11 (8.9)	10 (8.2)	11 (9.1)	3 (2.5)	

Variables	1st quintile (n=122)<br><br>n (%)	2nd quintile (n=123)<br><br>n (%)	3rd quintile (n=122)<br><br>n (%)	4th quintile (n=121)<br><br>n (%)	5th quintile (n=121)<br><br>n (%)	P-value*
Do you think communities should actively participate in controlling the vectors of DF?						0.017
Strongly agree	37 (30.3)	50 (40.7)	40 (33.1)	51 (42.1)	63 (52.1)	
Agree	50 (41.0)	41 (33.3)	48 (39.7)	39 (32.2)	40 (33.1)	
Not sure	16 (13.1)	15 (12.2)	5 (4.1)	8 (6.6)	5 (4.1)	
Disagree	12 (9.8)	8 (6.5)	17 (14.0)	11 (9.1)	6 (5.0)	
Strongly disagree	7 (5.7)	9 (7.3)	11 (9.1)	12 (9.9)	7 (5.8)	
Everyone has a chance to suffer from DF.						0.000
Strongly agree	28 (23.0)	29 (23.6)	28 (23.0)	36 (29.8)	38 (31.4)	
Agree	41 (33.6)	66 (53.7)	55 (45.1)	51 (42.1)	56 (46.3)	
Not sure	31 (25.4)	9 (7.3)	9 (7.4)	8 (6.6)	12 (9.9)	
Disagree	13 10.7)	11 (8.9)	16 (13.1)	16 (13.2)	11 (9.1)	
Strongly disagree	9 (7.4)	8 (6.5)	14 (11.5)	10 (8.3)	4 (3.3)	
If I experience signs and symptoms of DF, I would immediately come to a Community Health Centre.						0.045
Strongly agree	51 (41.8)	50 (40.7)	39 (32.0)	56 (46.3)	62 (51.2)	
Agree	46 (37.7)	43 (35.0)	56 (45.9)	38 (31.4)	44 (36.4)	
Not sure	9 (7.4)	8 (6.5)	3 (2.5)	8 (6.6)	4 (3.3)	
Disagree	11 (9.0)	8 (6.5)	11 (9.0)	5 (4.1)	6 (5.0)	
Strongly disagree	5 (4.1)	14 (11.4)	13 (10.7)	14 (11.6)	5 (4.1)	
You are the key individuals in preventing DF.						0.402
Strongly agree	27 (22.1)	34 (27.6)	23 (18.9)	34 (28.1)	42 (34.7)	
Agree	52 (42.6)	48 (39.0)	51 (41.8)	47 (38.8)	50 (41.3)	
Not sure	23 (18.9)	20 (16.3)	25 (20.5)	20 (16.5)	18 (14.9)	
Disagree	15 (12.3)	19 (15.4)	16 (13.1)	14 (11.6)	8 (6.6)	
Strongly disagree	5 (4.1)	2 (1.6)	7 (5.7)	6 (5.0)	3 (2.5)	
All DF patients have the chance of a full recovery.						0.030
Strongly agree	38 (31.1)	43 (35.0)	35 (28.7)	49 (40.5)	50 (41.3)	
Agree	49 (40.2)	48 (39.0)	44 (36.1)	43 (35.5)	53 (43.8)	
Not sure	21 (17.2)	10 (8.1)	19 (15.6)	7 (5.8)	8 (6.6)	
Disagree	10 (8.2)	14 (11.4)	17 (13.9)	13 (10.7)	8 (6.6)	
Strongly disagree	4 (3.3)	8 (6.5)	7 (5.7)	9 (7.4)	2 (1.7)	
The government is doing their best to prevent DF.						0.117
Strongly agree	21 (17.2)	12 (9.8)	12 (9.8)	15 (12.4)	18 (14.9)	
Agree	36 (29.5)	28 (22.8)	29 (23.8)	36 (29.8)	47 (38.8)	
Not sure	41 (33.6)	57 (46.3)	49 (40.2)	37 (30.6)	37 (30.6)	

Variables	1st quintile (n=122)<br><br>n (%)	2nd quintile (n=123)<br><br>n (%)	3rd quintile (n=122)<br><br>n (%)	4th quintile (n=121)<br><br>n (%)	5th quintile (n=121)<br><br>n (%)	P-value*
Disagree	18 (14.8)	20 (16.3)	26 (21.3)	27 (22.3)	15 (12.4)	
Strongly disagree	6 (4.9)	6 (4.9)	6 (4.9)	6 (5.0)	4 (3.3)	
Your neighborhood is a DF high-risk area.						0.056
Strongly agree	12 (9.8)	8 (6.5)	15 (12.3)	14 (11.6)	20 (16.5)	
Agree	24 (19.7)	39 (31.7)	27 (22.1)	37 (30.6)	30 (24.8)	
Not sure	46 (37.7)	43 (35.0)	41 (33.6)	36 (29.8)	47 (38.8)	
Disagree	31 (25.4)	29 (23.6)	36 (29.5)	32 (26.4)	23 (19.0)	
Strongly disagree	9 (7.4)	4 (3.3)	3 (2.5)	2 (1.7)	1 (0.8)	
Community members are capable of preventing DF.						0.447
Strongly agree	21 (17.2)	30 (24.4)	24 (19.7)	26 (21.5)	37 (30.6)	
Agree	43 (35.2)	45 (36.6)	45 (36.9)	52 (43.0)	51 (42.1)	
Not sure	35 (28.7)	26 (21.1)	30 (24.6)	24 (19.8)	21 (17.4)	
Disagree	17 (13.9)	17 (13.8)	18 (14.8)	15 (12.4)	10 (8.3)	
Strongly disagree	6 (4.9)	5 (4,1)	5 (4.1)	4 (3.3)	2 (1.7)	
You are capable of preventing DF.						0.010
Strongly agree	22 (18.0)	14 (11.4)	22 (18.0)	20 (16.5)	33 (27.2)	
Agree	42 (34.4)	57 (46.3)	40 (32.8)	57 (47.1)	58 (47.9)	
Not sure	37 (30.3)	31 (25.2)	36 (29.5)	21 (17.4)	20 (16.5)	
Disagree	14 (11.5)	16 (13.0)	19 (15.6)	17 (14.0)	9 (7.4)	
Strongly disagree	7 (5.7)	5 (4.1)	5 (4.1)	6 (5.0)	1 (0.8)	
Government actions are needed for DF prevention.						0.054
Strongly agree	48 (39.3)	51 (41.5)	43 (35.2)	57 (47.1)	55 (45.5)	
Agree	46 (37.7)	47 (38.2)	48 (39.3)	38 (31.4)	51 (42.1)	
Not sure	10 (8.2)	1 (0.8)	5 (4.1)	3 (2.5)	3 (2.5)	
Disagree	9 (7.4)	13 (10.6)	10 (8.2)	9 (7.4)	9 (7.4)	
Strongly disagree	9 (7.4)	11 (8.9)	16 (13.1)	14 (11.6)	3 (2.5)	

*Analyzed by ANOVA, but Fisher`s exact test was used where more than 20% of the cells had expected cell counts less than five
