# Supplementary material for: Knowledge, attitude, and practice regarding dengue virus infection among inhabitants of Aceh, Indonesia: a cross-sectional study
Source: BMC Infect Dis. 2018 Feb 27;18:96. doi: 10.1186/s12879-018-3006-z (PMC5830327; doi:10.1186/s12879-018-3006-z)
Supplement: Supplementary file 4 — Distribution of good practice regarding dengue fever prevention among participant groups with different socioeconomic level. (PDF 101 kb) [file 12879_2018_3006_MOESM4_ESM.pdf]

Additional file 4 - Table. Distribution of good practice regarding dengue fever prevention among participant groups with different socioeconomic level

Variables	1st quintile (n=122) n (%)	2nd quintile (n=123) n (%)	3rd quintile (n=122) n (%)	4th quintile (n=121) n (%)	5th quintile (n=121) n (%)	P-value*
Use screened windows to reduce mosquitoes. Yes	70 (57.4)	87 (70.7)	94 (77.0)	99 (81.8)	100 (82.6)	0.000
Prevent water stagnation. Yes	104 (85.2)	104 (84.6)	107 (87.7)	105 (86.8)	107 (88.4)	0.893
Cut down bushes in the yard to reduce mosquitoes. Yes	108 (88.5)	110 (89.4)	114 (93.4)	115 (95.0)	109 (90.1)	0.310
Use mosquito eating fish to reduce mosquitoes. Yes	27 (22.1)	34 (27.6)	23 (18.9)	31 (25.6)	39 (32.3)	0.148
Cleaning of garbage/trash. Yes	114 (93.4)	113 (91.9)	115 (94.3)	116 (95.9)	117 (96.7)	0.491
Disposing of water holding containers such as tires, parts of automobiles, plastic bottles, and cracked pots. Yes	103 (84.4)	103 (83.7)	101 (82.8)	104 (86.0)	110 (90.9)	0.402
Use of fan to prevent mosquito biting. Yes	62 (50.8)	83 (67.5)	87 (71.3)	95 (78.5)	99 (81.8)	0.000
Covering body with clothes when working in the bush, farm or forest. Yes	96 (78.7)	98 (79.7)	100 (82.0)	106 (87.6)	110 (90.9)	0.041
Cover water containers in the home. Yes	98 (80.3)	100 (81.3)	97 (79.5)	100 (82.6)	112 (92.6)	0.043
Change the water plant containers in the house every week. Yes	89 (73.0)	107 (87.0)	108 (88.5)	110 (90.9)	113 (93.4)	0.000
Change the water in flower containers. Yes	55 (45.1)	74 (60.2)	69 (56.6)	80 (66.1)	83 (68.6)	0.002
Check the waste/garbage that can block the flow of water around the home. Yes	103 (84.4)	108 (87.8)	107 (87.7)	104 (86.0)	113 (93.4)	0.265
Participate in any of the anti-dengue campaigns in the community. Yes	41 (33.6)	42 (34.4)	42 (34.4)	44 (36.4)	64 (52.9)	0.008
Check and clean the drains/gutters/roofs before the rainy season. Yes	64 (52.5)	68 (55.3)	73 (59.8)	69 (57.0)	84 (69.4)	0.075
Use a bed net when sleeping during the day. Yes	43 (35.2)	28 (22.8)	24 (19.7)	32 (26.4)	42 (34.7)	0.018

Variables	1st quintile (n=122)<br><br>n (%)	2nd quintile (n=123)<br><br>n (%)	3rd quintile (n=122)<br><br>n (%)	4th quintile (n=121)<br><br>n (%)	5th quintile (n=121)<br><br>n (%)	P-value*
Frequently cleaning water filled containers and ditches around the house. More than once per week	45 (36.9)	57 (46.3)	65 (53.3)	59 (48.8)	69 (57.0)	0.000

*Analysis by ANOVA, but Fisher`s exact test was used where more than 20% of the cells had expected cell counts less than five.
